# Lidocaine Inhibited Tendon Cell Proliferation and Extracellular Matrix Production by Down Regulation of Cyclin A, CDK2, Type I and Type III Collagen Expression

**DOI:** 10.3390/ijms23158787

**Published:** 2022-08-07

**Authors:** Yen-Chia Chen, Hsiang-Ning Chang, Jong-Hwei Su Pang, Li-Ping Lin, Jing-Min Chen, Tung-Yang Yu, Wen-Chung Tsai

**Affiliations:** 1 Department of Physical Medicine and Rehabilitation, Chang Gung Memorial Hospital, Taoyuan City 33302, Taiwan; 2 Graduate Institute of Clinical Medical Sciences, Chang Gung University, Taoyuan City 33302, Taiwan; 3 School of Medicine, Chang Gung University, Taoyuan City 33302, Taiwan; 4 Center of Comprehensive Sports Medicine, Chang Gung Memorial Hospital, Taoyuan City 33302, Taiwan

**Keywords:** lidocaine, tendon cells, cell proliferation, cell cycle, cyclin A, CDK 2, p21, MMP-9

## Abstract

Lidocaine injection is a common treatment for tendon injuries. However, the evidence suggests that lidocaine is toxic to tendon cells. This study investigated the effects of lidocaine on cultured tendon cells, focusing on the molecular mechanisms underlying cell proliferation and extracellular matrix (ECM) production. Tendon cells cultured from rat Achilles tendons were treated with 0.5, 1.0, or 1.5 mg/mL lidocaine for 24 h. Cell proliferation was evaluated by Cell Counting Kit 8 (CCK-8) assay and bromodeoxyuridine (BrdU) assay. Cell apoptosis was assessed by Annexin V and propidium iodide (PI) stain. Cell cycle progression and cell mitosis were assessed through flow cytometry and immunofluorescence staining, respectively. The expression of cyclin E, cyclin A, cyclin-dependent kinase 2 (CDK2), p21, p27, p53, matrix metalloproteinases-2 (MMP-2), matrix metalloproteinases-9 (MMP-9), type I collagen, and type III collagen were examined through Western blotting, and the enzymatic activity of MMP-9 was determined through gelatin zymography. Lidocaine reduced cell proliferation and reduced G1/S transition and cell mitosis. Lidocaine did not have a significant negative effect on cell apoptosis. Lidocaine significantly inhibited cyclin A and CDK2 expression but promoted p21, p27, and p53 expression. Furthermore, the expression of MMP-2 and MMP-9 increased, whereas that of type I and type III collagen decreased. Lidocaine also increased the enzymatic activity of MMP-9. Our findings support the premise that lidocaine inhibits tendon cell proliferation by changing the expression of cell-cycle-related proteins and reduces ECM production by altering levels of MMPs and collagens.

## 1. Introduction

Tendon injuries are common among athletes as well as the general population. In general practice consultations, tendon disorders account for approximately 30% of reported musculoskeletal pain [1]; moreover, they are associated with considerable morbidity and disability.

Local anesthetics such as lidocaine and bupivacaine are used in the treatment of clinical tendinopathy, either on their own or in combination with steroids for peritendinous injections [2]. However, local anesthetics may cause cell damage, and their toxicity to myocytes [3], chondrocytes [4,5,6,7], neurons [8], and mesenchymal stem cells [9] has been established. From 2012 to 2016, mounting evidence on the toxicity of local anesthetics to tendon cells has been presented. In vitro studies have suggested that lidocaine [10,11,12,13] and bupivacaine [13,14] are toxic to tendon cells. Specifically, lidocaine negatively affected cell viability and proliferation, and the researchers indicated that such local anesthetics should be administered with caution [10,12]. However, few studies have discussed the molecular mechanisms of lidocaine in the tenocyte cell cycle or in related kinases.

The structure of a tendon mainly consists of tendon cells, collagen fibrils, and the surrounding extracellular matrix (ECM) [15]. Cell proliferation, collagen production, and ECM remodeling are crucial in the tendon healing process. First, cell proliferation occurs following the cell cycle. Comprising the G0/G1, S, G2, and M phases, the cell cycle is precisely regulated by various cyclin-dependent kinases (CDKs) and their partnering cyclins [16]. Some researchers have identified the accumulation of cyclins and CDKs as the principal regulator of the G1/S transition and of the progression of remaining cell cycles [17,18]. In the later G1 phase, the level of cyclin E increases, and cyclin E–CDK2 complexes are formed, constituting a key step in the G1/S transition. CDK2 is a critical partner of cyclin A, and the activation of cyclin A–CDK2 complexes is essential for S-phase progression [17,18].

The formation of collagen fibrils, fundamental units of tendons, is a key factor in tendon repair [19]. In uninjured tendons, approximately 90% and <10% of collagen is type I and type III, respectively [20]. Tendon cells produce type I collagen and associated ECM molecules [15], contributing to the homeostasis of the ECM and maintaining a constant equilibrium between synthesis and degradation [21]. Matrix metalloproteinases (MMPs), a group of ECM-degrading enzymes, are crucial regulators of ECM network remodeling [22,23]. Studies have reported that MMP levels are altered during tendon healing [19,22]. MMP-2 and MMP-9 have specifically been associated with collagenolytic activity [19,24].

To gain an in-depth understanding of how lidocaine may affect tendon healing on a molecular level, we investigated the effects of this anesthetic on cultured tendon cells, focusing on the molecular mechanisms underlying cell proliferation and collagen synthesis.

## 2. Results

### 2.1. Lidocaine Inhibited Cell Proliferation in an Ex Vivo Tendon Explant Model

**Figure 1:**
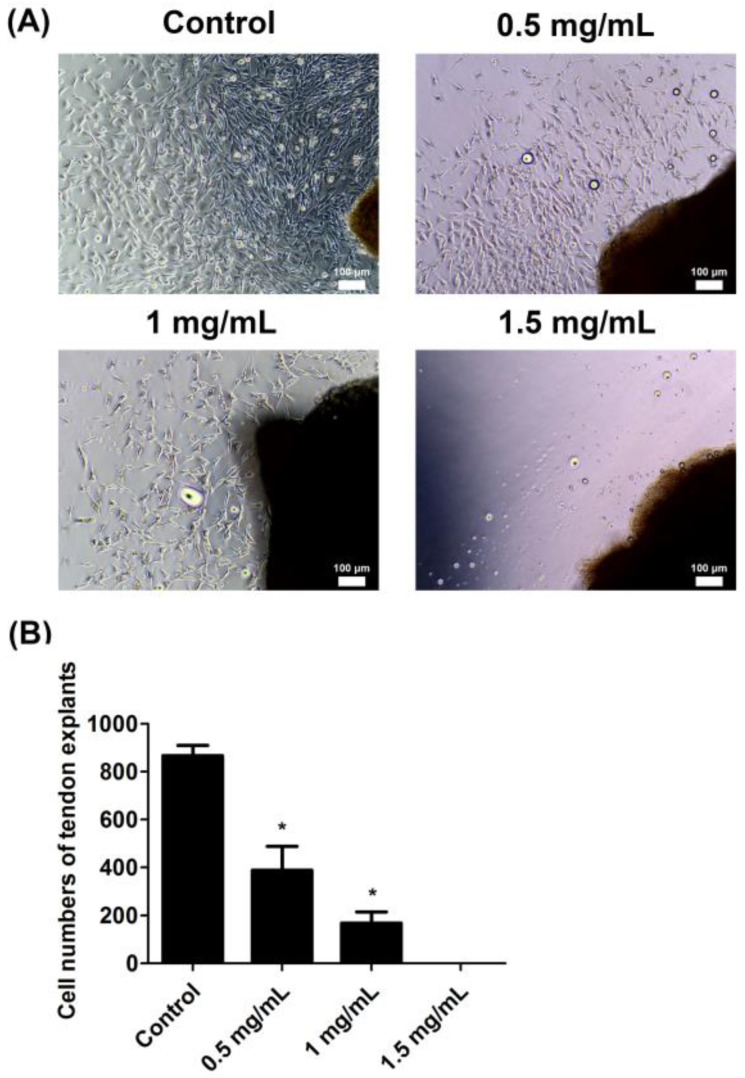
Lidocaine reduced tendon cell proliferation ex vivo. (**A**) Tendon explants were seeded in culture plates and were either left untreated or were treated with lidocaine (0.5, 1.0, or 1.5 mg/mL). (**B**) The cell numbers of the tendon explant in the control, 0.5, 1.0, or 1.5 mg/mL groups. Data are presented as the means ± standard errors of the mean of three independent experiments. * *p* < 0.05.

An ex vivo culture of tendons was used to assess the effect of lidocaine on tendon cell proliferation. The tendon explants were seeded in a culture dish and left untreated or treated with different concentrations of lidocaine (0.5 mg/mL, 1.0 mg/mL, and 1.5 mg/mL). At 4 days post seeding, the cells migrated out of the tendon explants and proliferated (Figure 1A). The cell number of each tendon explant was counted (Figure 1B). The results documented that lidocaine inhibited cell proliferation in an ex vivo tendon explant model.

### 2.2. Lidocaine Reduced Tendon Cell Proliferation

**Figure 2:**
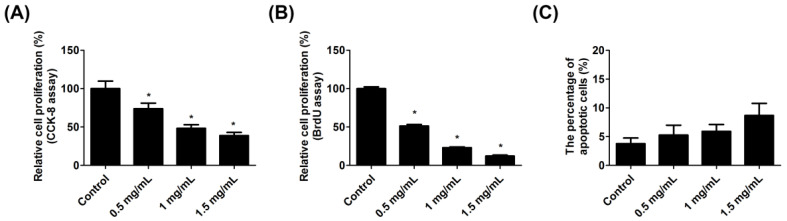
Lidocaine reduced tendon cell proliferation. Tendon cells were either left untreated or were treated with lidocaine (0.5, 1.0, or 1.5 mg/mL) for 24 h. (**A**) The cell viability was determined through the CCK-8 assay. (**B**) The cell proliferation was determined by BrdU assay. (**C**) The cell apoptosis was determined by Annexin V and propidium iodide (PI) stain. Data are presented as the means ± standard errors of the mean of three independent experiments. * *p* < 0.05.

The Cell Counting Kit 8 (CCK-8) assay revealed that the cell viability of tendon cells decreased after lidocaine treatment. The relative cell viabilities are listed as follows: 100.0 ± 9.8% in the control group, 73.8 ± 7.3% in the 0.5 mg/mL lidocaine group, 48.2 ± 4.8% in the 1.0 mg/mL lidocaine group, and 38.8 ± 4.1% in the 1.5 mg/mL lidocaine group (Figure 2A). The Bromodeoxyuridine (BrdU) assay revealed that the cell proliferation of tendon cells decreased after lidocaine treatment. The relative cell proliferation ability in the control group was 100.0 ± 2.3%. In the 0.5 mg/mL, 1.0 mg/mL, and 1.5 mg/mL lidocaine groups, the corresponding percentages were 51.4 ± 1.9%, 23.0 ± 1.1%, and 12.0 ± 1.3%, respectively (Figure 2B). Furthermore, cell apoptosis was detected by using Annexin V and propidium iodide (PI) stain. The percentage of apoptotic cells was 3.8 ± 1.0% in control group and 5.3 ± 1.7%, 5.9 ± 1.2%, and 8.7 ± 2.1% in cells treated with 0.5, 1.0, or 1.5 mg/mL lidocaine, respectively. There were no statistically significant differences between the control group and the lidocaine-treated group (Figure 2C).

### 2.3. Lidocaine Inhibited S-Phase Progression

**Figure 3:**
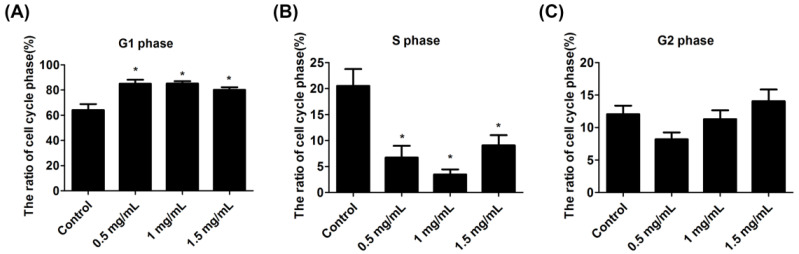
Cell cycle analysis of tendon cells. Tendon cells were either left untreated or were treated with 0.5 mg/mL, 1.0 mg/mL, or 1.5 mg/mL lidocaine for 24 h. The cell cycle analysis was performed by flow cytometry. The result for the G1 phase is presented in (**A**). The result for the S phase is presented in (**B**). The result for the G2 phase is presented in (**C**). Data are presented as the means ± standard errors of the mean of three independent experiments. * *p* < 0.05.

Flow cytometry revealed that lidocaine lengthened the G1 phase and shortened the S phase of tendon cells. The percentage of cells in the G1 phase in the control group was 64.1 ± 4.7%. In the 0.5 mg/mL, 1.0 mg/mL, and 1.5 mg/mL lidocaine groups, the corresponding percentages were 85.1 ± 3.1%, 85.2 ± 1.8%, and 80.2 ± 2.1%, respectively (Figure 3A). The percentage of cells in the S phase in the control group was 20.53 ± 3.22%, and the corresponding percentages in the 0.5 mg/mL, 1.0 mg/mL, and 1.5 mg/mL lidocaine groups were 6.72 ± 2.26%, 3.46 ± 0.99%, and 9.07 ± 1.96%, respectively (Figure 3B). No significant changes in the proportions of cells in the G2 phase were observed in any group (Figure 3C).

### 2.4. Lidocaine Inhibited Mitosis in Tendon Cells

**Figure 4:**
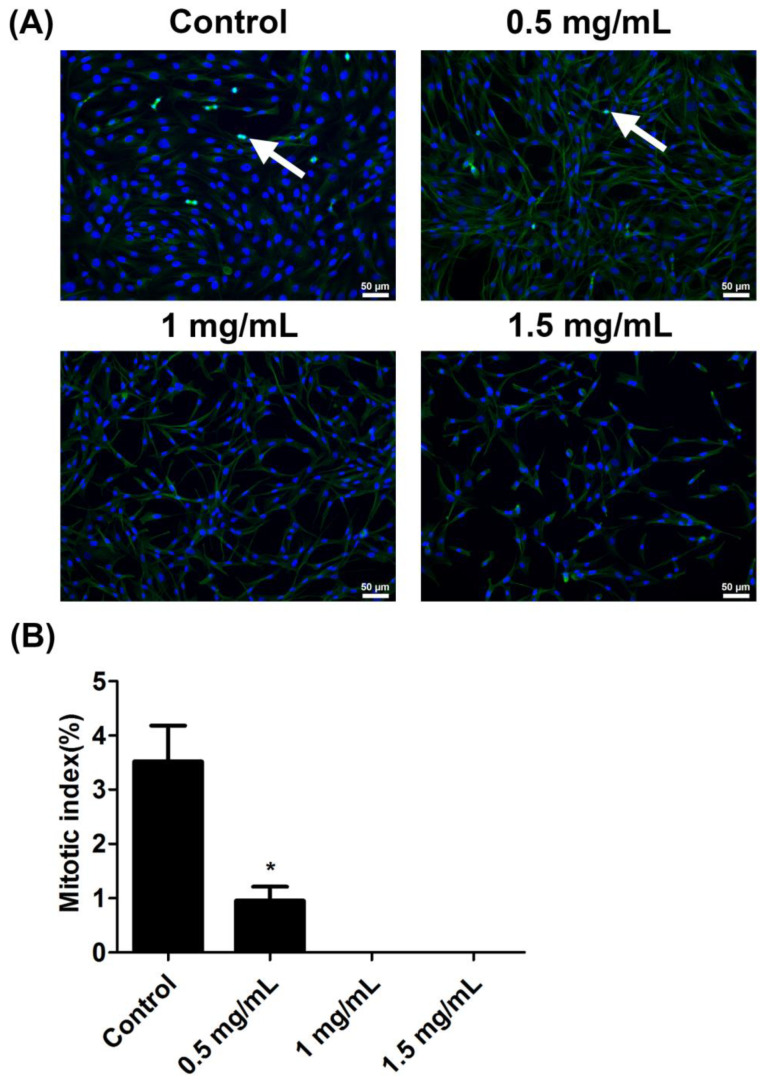
Lidocaine inhibited mitosis in tendon cells. (**A**) Tendon cells were either left untreated or were treated with 0.5, 1.0, or 1.5 mg/mL lidocaine for 24 h, after which mitosis was observed through immunofluorescence staining. Tubulin was stained green, and nuclei were stained blue. White arrows point to mitotic cells. Scale bar: 50 μm. (**B**) The numbers of mitotic cells and total cells were counted. The mitotic index was defined as the number of nuclei undergoing mitosis divided by the number of all cells on the coverslip and then multiplied by 100%. Data are presented as the means ± standard errors of the mean of three independent experiments. * *p* < 0.05.

The mitosis of cells was determined by immunofluorescence staining (Figure 4A). The result revealed that lidocaine significantly reduced the mitotic index in the 0.5 mg/mL lidocaine group. Notably, no mitotic cells were found in the 1.0 and 1.5 mg/mL lidocaine group (Figure 4B).

### 2.5. Lidocaine Reduced the Expression of Cyclin A, CDK1, and CDK2 via the p21, p27, and p53 Pathways

**Figure 5:**
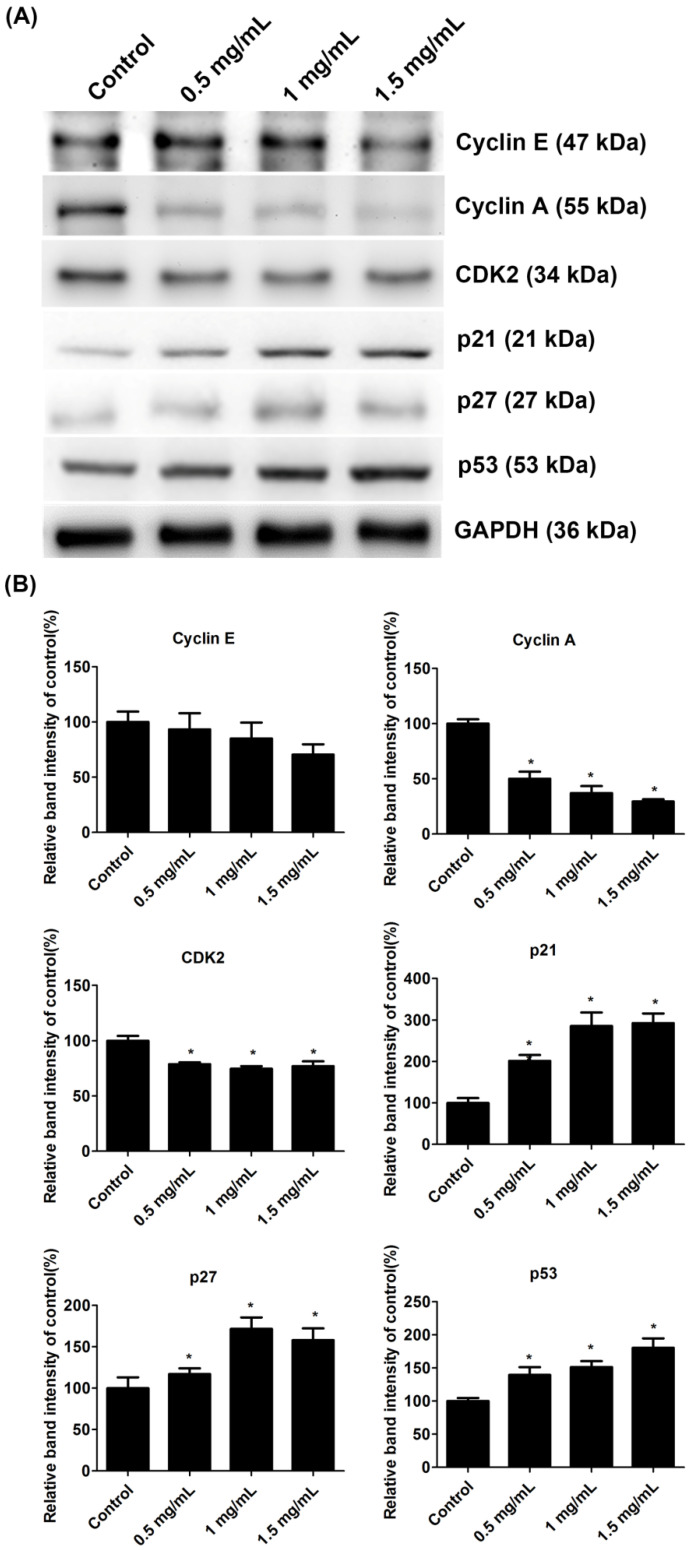
Lidocaine regulated the expression of cell-cycle-associated proteins. (**A**) Western blot analysis of cell cycle–associated proteins. The glyceraldehyde 3-phosphate dehydrogenase (GAPDH) was used as the internal control. (**B**) Densitometric analysis of cyclin E, cyclin A, CDK2, p21, p27, and p53. All data were normalized to GAPDH. Data are presented as the means ± standard errors of the mean of three independent experiments. * *p* < 0.05.

The cell-cycle-regulated proteins of lidocaine-treated tendon cells were examined. Lidocaine inhibited the expression of cyclin A and CDK2 but promoted the expression of p21, p27, and p53 (Figure 5A). However, the expression of cyclin E exhibited no significant changes in any group. The band intensity of cyclin E was 100.0 ± 9.4% in the control group and 93.3 ± 14.6%, 85.0 ± 14.5%, and 70.5 ± 9.3% in cells treated with 0.5, 1.0, or 1.5 mg/mL lidocaine, respectively. The band intensity of cyclin A was 100.0 ± 4.0% in the control group and 50.1 ± 6.4%, 37.1 ± 6.3%, and 29.5 ± 2.0% in cells treated with 0.5, 1.0, or 1.5 mg/mL lidocaine, respectively. The band intensity of CDK2 was 100.0 ± 4.4% in the control group and 78.9 ± 1.7%, 74.6 ± 2.4%, and 77.1 ± 4.3% in cells treated with 0.5, 1.0, or 1.5 mg/mL lidocaine, respectively. The band intensity of p21 was 100.0 ± 11.8% in the control group and 201.5 ± 14.3%, 285.7 ± 32.8%, and 292.7 ± 23.3% in cells treated with 0.5, 1.0, or 1.5 mg/mL lidocaine, respectively. The band intensity of p27 was 100.0 ± 13.1% in the control group, and 116.9 ± 7.0%, 171.5 ± 13.9%, and 157.8 ± 14.4% in cells treated with 0.5, 1.0, or 1.5 mg/mL lidocaine, respectively. The band intensity of p53 was 100.0 ± 4.5% in the control group and 139.5 ± 11.5%, 151.4 ± 8.9%, and 180.4 ± 14.4% in cells treated with 0.5, 1.0, or 1.5 mg/mL lidocaine, respectively (Figure 5B).

### 2.6. Lidocaine Increased the Enzymatic Activities of MMP-9

**Figure 6:**
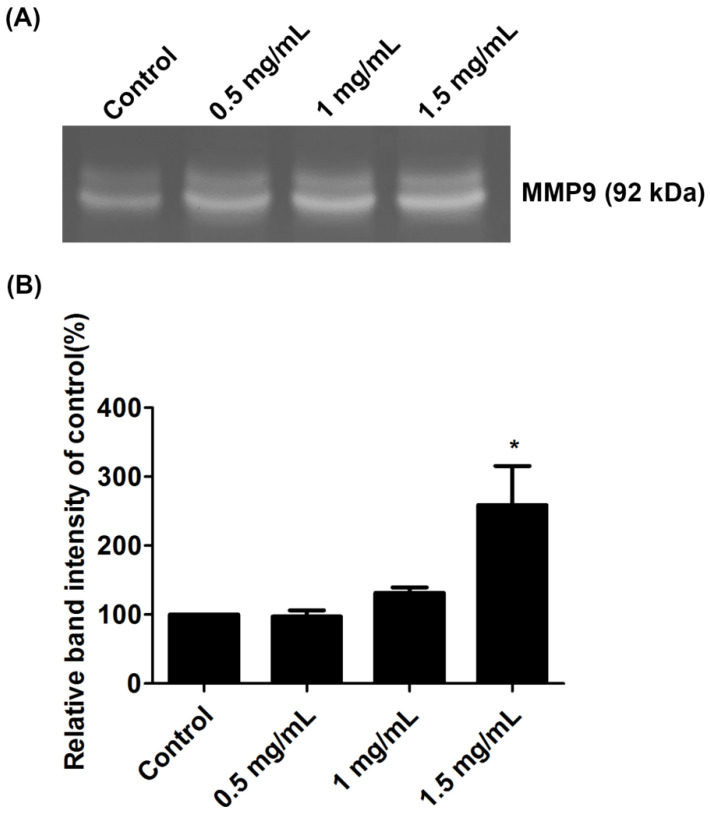
Gelatin zymography of tendon cells involving the (**A**) enzymatic activities of MMP-9 in conditioned medium and (**B**) densitometric analysis of MMP-9. Data are presented as the means ± standard errors of the mean of three independent experiments. * *p* < 0.05.

Gelatin zymography analysis demonstrated that the MMP-9/gelatinase B activity of tendon cells was induced by lidocaine (Figure 6A). Densitometric analysis showed that MMP-9 activity under the high-dose lidocaine treatment (1.5 mg/mL) was significantly higher than that under the control conditions (*p* < 0.05; Figure 6B).

### 2.7. Lidocaine Promoted MMP Expression and Suppressed Collagen Production

**Figure 7:**
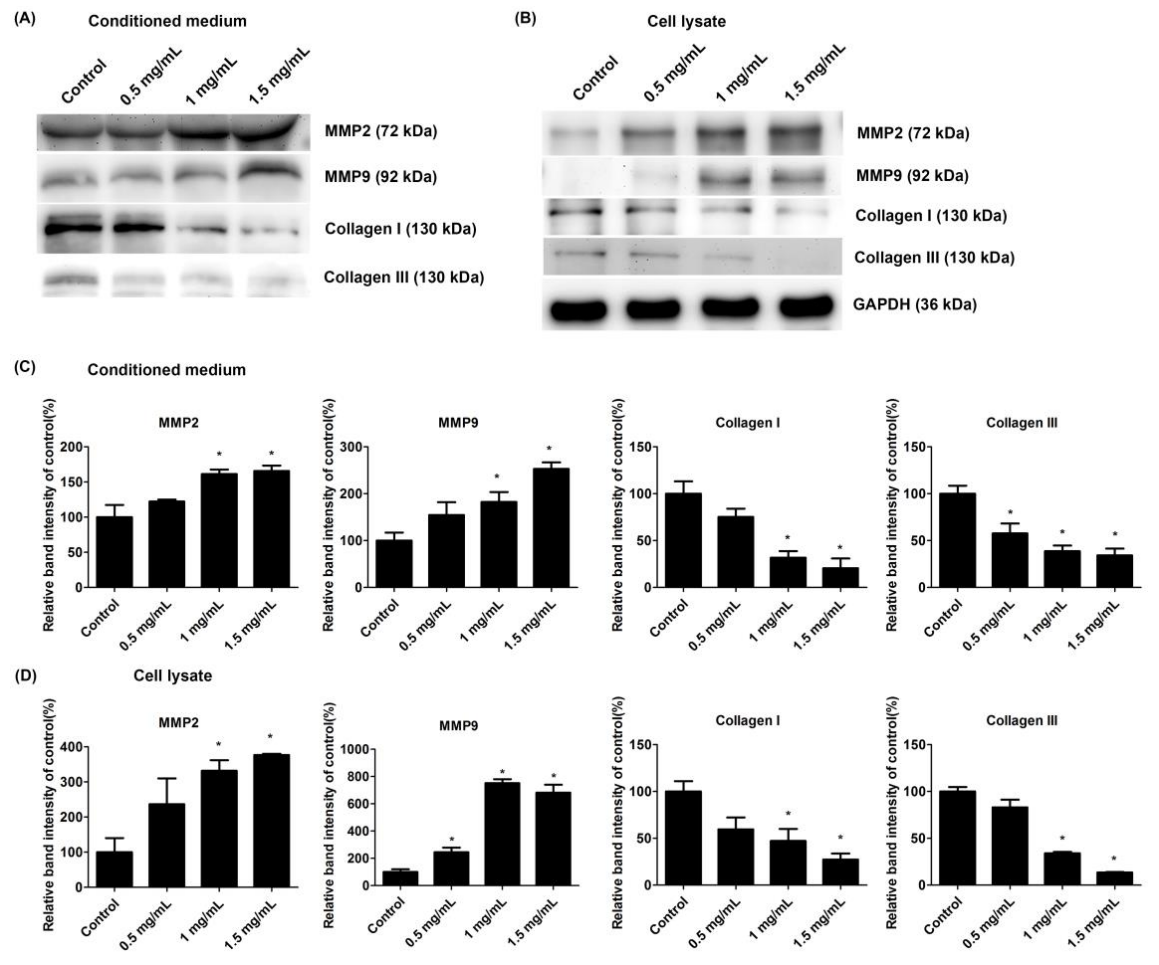
Lidocaine changed MMP expression and altered collagen production in tendon cells. Western blot analysis of the expression of MMP-2, MMP-9, type I collagen, and type III collagen in (**A**) conditioned medium and (**B**) cell lysate. (**C**,**D**) Densitometric analysis of MMP-2, MMP-9, type I collagen, and type III collagen. The loading volume of the conditioned medium was adjusted by the number of cells in the control, 0.5 mg/mL, 1.0 mg/mL, and 1.5 mg/mL groups. The GAPDH was used as the internal control in the cell lysate group. The data of the densitometric analysis of were normalized to the GAPDH in cell lysate group. Data are presented as the means ± standard errors of the mean of three independent experiments. * *p* < 0.05.

We investigated the expression of MMPs and collagen in tendon cells treated with 0.5 mg/mL, 1.0 mg/mL, and 1.5 mg/mL lidocaine. Western blotting of the conditioned medium and cell lysate revealed that lidocaine promoted the expression of MMP-2 and MMP-9 (Figure 7A,B). Significant increases in the expression of MMP-2 and MMP-9 were observed in the densitometric analysis (Figure 7C). However, lidocaine reduced both secreted and cytosolic collagen (type I and type III) in the tendon cells (Figure 7A,B). Densitometric analysis also indicated significant declines in type I and type III collagen (Figure 7C,D).

## 3. Discussion

Local anesthetics are commonly injected into injured tendons in clinical settings. In vitro studies have reported the significant toxicity of local anesthetics to mesenchymal stem cells [9,25], neurons [8], myocytes [3], and chondrocytes [4,5,6,7], among other cell types. More recent studies have revealed that the administration of lidocaine to cultured tendon cells exerted detrimental effects on cell viability [10,11,12]. One investigation determined that lidocaine reduces the gene expression of scleraxis and type I collagen, which are essential for early tenogenic differentiation and tendon healing [12]. An animal study indicated that lidocaine treatment for tendon injury reduces the biomechanical strength of the tendon and suppresses the production of collagen [10]. Sung et al. found that local anesthetics classified as aminoamide compounds induce cell death by promoting the intracellular generation of reactive oxygen species and the activities of mitogen-activated protein kinase and caspase-3/7 [13]. Growing evidence suggests that such agents commonly used to treat tendon injuries may actually inhibit tendon healing.

The process of tendon healing consists of three overlapping phases: the Inflammatory phase, the proliferative (reparative) phase, and the remodeling phase [26,27]. The initial inflammatory phase begins shortly after injury, with white blood cells attracted to the site by cytokines and initiating vascular formation [28]. A few days later, fibroblast recruitment occurs, and the proliferative phase begins. This phase is characterized by an increase in cellularity and the synthesis of abundant ECM components, mainly proteoglycans and type III collagen [27]. In the remodeling phase, which commences 6–8 weeks after injury, cellularity and matrix production decrease, and type I collagen replaces type III collagen. Collagen fibers start forming arrangements, and the tendonous tissue matures [27]. During all these phases, but especially during the proliferative phase, tendon cell proliferation and migration are essential, as are collagen synthesis and ECM production [19,26].

Cell proliferation is regulated by the cell cycle, which comprises a series of events leading to cell division. The progression of the cell cycle is regulated by the coordinated activities of cyclin–CDK complexes [16]. Herein, significantly fewer lidocaine-treated tendon cells than untreated tendon cells entered the S phase, indicating that lidocaine causes cell cycle arrest; it prevents tendon cells from advancing from the G1 phase to the S phase. This observation may be attributable to the reduced expression of cyclin A and CDK2 in the lidocaine-treated groups. Through densitometric analysis, we determined that the expression of cyclin A and CDK2 decreased dose-dependently with lidocaine treatment. The formation and activation of cyclin E–CDK2 complexes and cyclin A–CDK2/1 complexes are critical for the progression of cells through the G1 phase, the G1/S transition, and the S phase [17,18,29]. We also detected significant increases in the expression of p21, p27, and p53 in the lidocaine-treated group. Members of the CIP/KIP family, p21 and p27 are CDK inhibitors shown to be capable of controlling CDK activities [30,31]. p21 and p27 inhibit the G1/S transition by binding to cyclin A–CDK2, cyclin E–CDK2, and cyclin D–CDK4 complexes [29,31]. Notably, p21 is a transcriptional target of the tumor suppressor gene p53, which mediates cell cycle checkpoints. The increased expression of p53 prevents the progression of the cell cycle [32]. Thus, the observed decreases in the expression of cyclin A and CDK2 and the observed increases in the expression of p21, p27, and p53 suggest that lidocaine suppresses the proliferation of tendon cells by blocking their progression through the cell cycle.

ECM homeostasis is vital during both the proliferative and remodeling phases of tendon healing. Tendon cells both synthesize and degrade ECM components during tendon repair and remodeling [19,33]. MMPs participate in collagen degradation and remodeling and thus are pivotal regulators of the ECM network [19]. Research has linked the expression of certain MMPs with tendon injury and tendon rupture. Studies have reported that the expression of MMP-2 and MMP-9 is higher in injured tendons than in healthy tendons [23,34]. The increased expression of both MMP-2 and MMP-9 in the lidocaine-treated groups suggests that lidocaine enhances collagenolytic activity during ECM remodeling. These increases were detected in both cell lysate and conditioned medium, reinforcing the finding that lidocaine changed the ECM environment. Gelatin zymography provided further evidence of the elevated enzymatic activity of MMP-9 in the 1.5 mg/mL lidocaine group. These changes are likely to induce collagen degradation, which can compromise the integrity of the ECM of a tendon. This postulation is supported by the reduced expression of both type I and type III collagen in the lidocaine-treated groups. Collagen fibrils are regarded as the fundamental unit from which the tendon transmits force [15]. Thus, the reduced expression of type I and type III collagen in the treatment groups suggests that collagen production is suppressed by lidocaine, leading to the same concern that lidocaine impedes tendons from healing from injury.

Few studies on lidocaine and tendon healing have discussed the molecular mechanisms by which lidocaine exerts toxicity in tendon cells. One study described the impact of lidocaine on the cell cycle and the expression of cyclins and CDKs in melanoma cells [35], but ours is the first to confirm such effects on tendon cells. Furthermore, to the best of our knowledge, the current study is the first to document how lidocaine affects the expression of MMPs and collagen, as well as the enzymatic activity of MMP-9, in tendon cells. Lidocaine significantly reduced tendon cell proliferation, inhibited the expression of proteins related to cell cycles, increased MMP expression, and suppressed collagen production. These results are consistent with those of previous studies [10,11,12,13] but provide additional information regarding the molecular mechanisms through which lidocaine potentially hinders tendons from healing. This study has several limitations. First, given the in vitro nature of this study, researchers should be cautious when applying the findings to in vivo conditions. In order to justify the importance for further in vivo study, we performed ex vivo cultures of tendons to assess the effect of lidocaine on tendon cell proliferation, and the results indicated that lidocaine inhibited cell proliferation in an ex vivo tendon explant model. Second, the range of lidocaine concentrations tested was limited, and determining whether these concentrations are representative of those employed in peritendinous injections in clinical settings is challenging. The most common concentration of lidocaine used in clinical settings is a 1% (10 mg/mL) or 2% solution, which is much higher than the concentrations used in this study (i.e., 0.5, 1.0, and 1.5 mg/mL) [2]. However, the lidocaine concentration in extracellular matrix surrounding the tendon cells after a peritendinous injection has never been reported. Regarding the time exposure effect, the cells were left untreated or treated with lidocaine for 24 h or 48 h, and the cell viability was determined by CCK-8 assay. The results showed similar trends in tendon cells treated with lidocaine for 24 h or 48 h (Supplementary Figure S1). However, the true time of exposure for intratendinous and peritendinous injections in clinical settings remains difficult to predict. Under clinical conditions, the duration and concentration of cellular exposure is affected by multiple factors, including diffusion, dilution, and the metabolism of the injected agents by the body part in question. Significant variation may occur depending on the body parts involved and the inflammatory status of the injury (e.g., acute Achilles tendon tear versus chronic rotator cuff tendinopathy). Further in vitro and in vivo studies are warranted to determine whether the observed cytotoxicity of lidocaine has a clinically meaningful impact on tendon injuries. In conclusion, lidocaine inhibited tendon cell proliferation by reducing the expression of cyclin A and CDK2 and by increasing the expression of p21, p27, and p53, thus impeding cell cycle progression. Moreover, lidocaine affected the ECM production of tendon cells by increasing the expression of MMP-2 and MMP-9 and suppressing the expression of type I and type III collagen. These findings partly explain the molecular mechanisms through which lidocaine exerts toxicity in tendon cells. In addition, these findings provide evidence supporting the premise that lidocaine may hinder the healing of injured tendons.

## 4. Materials and Methods

### 4.1. Primary Culture of Rat Achilles Tendon Cells

Achilles tendons were obtained from Sprague Dawley rats, male, weighing 200 to 250 g, which were obtained from BioLASCO Taiwan Co.,Ltd. Taipei, Taiwan. The excised tendons were cut into small pieces (1.5–2.0 mm^3^) and seeded in 10-cm dishes. After 5 min of air-drying to improve adherence, the explants were cultured in a solution of Dulbecco’s modified Eagle’s medium (Thermo Fisher Scientific, Waltham, MA, USA), 20% fetal bovine serum (Thermo Fisher Scientific, Waltham, MA, USA), and 100 U/mL penicillin/100 μg/mL streptomycin (Gibco, 15140122, Thermo Fisher Scientific, Waltham, MA, USA) at 37 °C in a humidified atmosphere of 5% CO_2_/95% air. After 2 to 3 days, the cells started to migrate from the explants. These tendon cells were used in subsequent studies.

### 4.2. Ex Vivo Tendon Explant Model

The excised tendons were cut into small pieces (1.5–2.0 mm^3^) and seeded on 6-well plates. The explants were left untreated or treated with different concentrations of lidocaine (0.5, 1.0, and 1.5 mg/mL). After 4 days, the cells migrated from the tendon explants. The cell number of each tendon explant was counted.

### 4.3. Cell Counting Kit 8 (CCK-8) Assay

Tendon cells were seeded into 24-well plates (1 × 10^4^ cells/well) and treated with various concentrations of lidocaine (0.5, 1.0, and 1.5 mg/mL) for 24 h. Cell proliferation was detected using the Cell Counting Kit 8 (CCK-8; BIOTOOLS Co., Ltd., Taipei, Taiwan). CCK-8 reagent was added to each well, which was then incubated at 37 °C in a humidified atmosphere of 5% CO_2_/95% air for 1 h. The absorbance at 450 nm was measured using a multiwell spectrophotometer (SpectraMax iD3, Molecular Devices, LLC., San Jose, CA, USA).

### 4.4. Bromodeoxyuridine (BrdU) Assay

BrdU assay was performed according to the manufacturer’s protocol. Briefly, the cells were labeled with BrdU reagent (Roche). After fixing and washing, the substrate solution and stop solution were added to each well. The absorbance at 450 nm was measured using a multiwell spectrophotometer (SpectraMax iD3, Molecular Devices, LLC., San Jose, CA, USA).

### 4.5. Apoptosis Assay

The apoptotic cells were determined using an Annexin V-FITC Apoptosis Detection Kit in accordance with the manufacturer’s protocol (Strong Biotech Corporation, Taipei, Taiwan). Briefly, cells were collected and stained with Annexin V reagent and propidium iodide (PI) reagent. The analysis was performed using the Becton Dickinson FACScan System (San Francisco, CA, USA).

### 4.6. Cell Cycle Analysis

The lidocaine-treated tendon cells were washed twice with phosphate-buffered saline (PBS) and fixed with 70% ethanol in PBS for 1 h at −20 °C. Following centrifugation at 3000 RPM for 3 min, the cells were suspended in 1 mL of PBS containing 0.5% Triton X-100 and 0.05% RNase A. Subsequently, they were incubated at 37 °C for 1 h and then washed with PBS. The cells were then resuspended in PBS containing 20 μg/mL propidium iodide and incubated for 20 min at 4 °C. Next, flow cytometry analysis was conducted using the Becton Dickinson FACScan System (San Francisco, CA, USA).

### 4.7. Immunofluorescence Staining

For immunofluorescence staining, tendon cells were treated with lidocaine for 24 h and then fixed in 10% formalin for 15 min. The cells were washed in PBS three times and incubated in blocking solution (Thermo Fisher Scientific, Waltham, MA, USA) for 30 min. Subsequently, they were incubated for 2 h with an anti-tubulin antibody (Thermo Fisher Scientific, Waltham, MA, USA) diluted in blocking solution. The signal was detected using anti-mouse immunoglobulin (IgG) Alexa Fluor 488 (Thermo Fisher Scientific, Waltham, MA, USA). After being washed with PBS, the cells were mounted with mounting buffer containing 4′,6-diamidino-2-phenylindole (Abcam, Cambridge, MA, USA). The cells were observed under a fluorescence microscope at 200× magnification (Eclipse Ni-U; Nikon, Tokyo, Japan). The mitotic index was defined as the number of nuclei undergoing mitosis divided by the number of all cells on the coverslip and then multiplied by 100%.

### 4.8. Gelatin Zymography

MMP-9 activity in the conditioned medium was detected through gelatin zymography. The sample was examined under nonreducing conditions by using an 8% sodium dodecyl sulfate (SDS) polyacrylamide gel containing 1 mg/mL gelatin. The gel was washed in 2.5% Triton X-100 to remove SDS and to allow MMP renaturation. Moreover, the gel was incubated with reaction buffer (0.2 M NaCl, 50 mM Tris [pH 7.5], 5 mM CaCl_2_, and 0.02% BRIJ^®^ 35) at 37 °C for 48 h, after which it was stained with Coomassie Brilliant Blue R-250 Staining Solution (Bio-Rad Laboratories, Hercules, CA, USA). The presence of pro-MMPs and active MMPs resulted in white lysis bands attributable to gelatin degradation.

### 4.9. Western Blot Analysis

Cell extracts were prepared in a lysis buffer containing 1 mM EDTA, 1 mM EGTA, 1 mM DTT, 0.5 mM PMSF, 1 mM Na_3_VO_4_, 1 mM Na_2_P_2_O_7_, 20 mM NaF, 20 mM HEPES, 1 μg/mL leupeptin, and 1% Triton X-100. The protein concentration of the cell extracts was determined through Bradford protein assays. Samples with identical quantities of protein were separated by 10% SDS polyacrylamide gel electrophoresis and transferred onto a PVDF membrane. The membranes were incubated at room temperature in blocking solution (5% BSA in Tris-buffered saline containing 0.1% Tween 20 [TBST]) for 1 h. This was followed by 2 h incubation in blocking solution containing an appropriate dilution of a primary antibody, such as anti-glyceraldehyde 3-phosphate dehydrogenase (GAPDH; Proteintech Group, Inc., Rosemont, IL, USA) and anti-cyclin E, anti-cyclin A, anti-CDK2 (ABclonal, Cambridge, MA, USA), anti-p21, anti-p53 (Thermo Fisher Scientific, Waltham, MA, USA), anti-p27 (Cell Signaling Technology, Danvers, MA, USA), anti-MMP-2 (Proteintech Group, Inc., Rosemont, IL, USA), anti-MMP-9 (Abcam), anti-type-I-collagen, or anti-type-III-collagen antibodies (Proteintech Group, Inc., Rosemont, IL, USA). The membranes were washed in TBST and then incubated in TBS containing anti-mouse IgG conjugated with horseradish peroxidase (Leinco Technologies, Inc., St. Louis, MO, USA) or antirabbit IgG conjugated with horseradish peroxidase (Cell Signaling Technology, Danvers, MA, USA) for 1 h. Next, the membranes were washed three times in TBST and developed on a Luminata Crescendo Western HRP substrate (Merck Millipore, Darmstadt, Germany). Band intensities were analyzed using ImageJ software. GAPDH was used as an internal control. The experiment was performed in triplicate.

### 4.10. Statistical Analysis

All data are expressed as means ± standard errors of the mean. All experiments were performed in triplicate. The Kruskal–Wallis test was performed for group comparisons, and the Mann–Whitney test was conducted to identify where between-group differences occurred. Differences were considered significant at *p* < 0.05.

## Data Availability

Data used for this study is available on request from the corresponding authors.

## Supplementary Materials

The following supporting information can be downloaded at: https://www.mdpi.com/article/10.3390/ijms23158787/s1.
