# Supplementary material for: Lidocaine Inhibited Tendon Cell Proliferation and Extracellular Matrix Production by Down Regulation of Cyclin A, CDK2, Type I and Type III Collagen Expression
Source: Int J Mol Sci. 2022 Aug 7;23(15):8787. doi: 10.3390/ijms23158787 (PMC9368801; doi:10.3390/ijms23158787)
Supplement: Supplementary file 1 [file ijms-23-08787-s001.zip › ijms-1859219-supplementary.pdf]

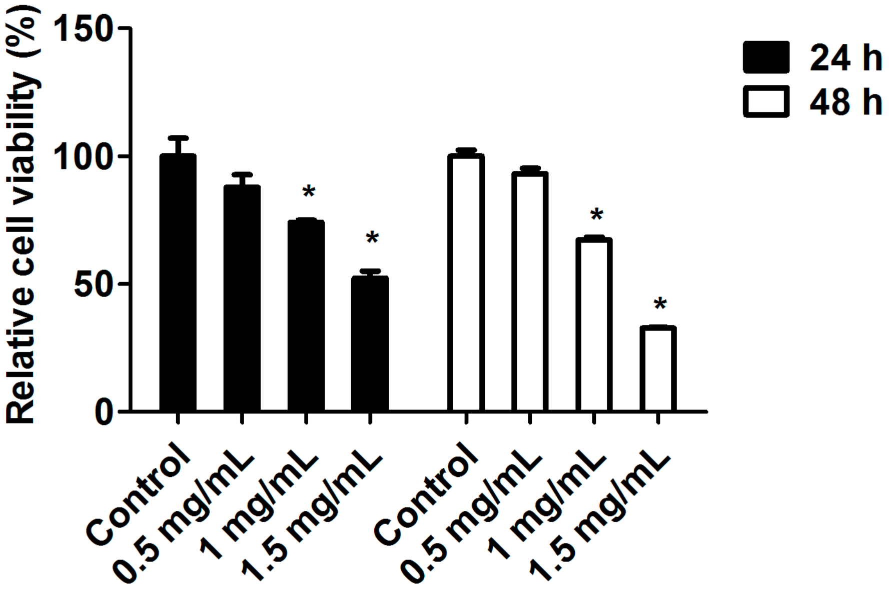

Figure S1. Cell viability was reduced by lidocaine treatment for 24 h and 48 h. Tendon cells were treated with lidocaine for 24 h and 48 h, and cell viability was performed by CCK-8 assay. Data are presented as the means ± standard errors of the mean of three independent experiments.*$p < 0.05$.
